# The Systematic Removal of Participants Post-Randomization Can Lead to Alternate Explanations of the Results

**DOI:** 10.2196/jmir.2631

**Published:** 2013-06-24

**Authors:** John A Cunningham

**Affiliations:** ^1^Centre for Mental Health ResearchThe Australian National UniversityCanberraAustralia; ^2^Centre for Addiction and Mental HealthToronto, ONCanada

**Keywords:** research methods, randomized controlled trial, confound, alternate explanation

The strength of randomized controlled trials is that they allow causal statements to be made about the efficacy of an intervention. This is because the randomization of participants to experimental condition distributes participants with different characteristics to each experimental condition by chance, including variations in the outcome variables of interest. Such randomization allows the use of statistical tests incorporating probability statements regarding the chance that the difference observed between conditions is due to chance (eg, 1 in 20 chance; *P*<.05).

Any systematic removal of participants post-randomization interferes with the assumption of the randomness of participant allocation to experimental condition. This can introduce a potential confound or alternative explanation of the results. The recently published study by Rooke et al [1] removed participants from the analysis who reported other treatment use during the study (n=5). The removal of participants was done post-randomization and in a systematic fashion (ie, anyone who reported using other treatment was not included in the analysis). All participants who reported receiving other treatment were in the control condition and were allocated to receive the Web address of an education only website. Even if some participants in the intervention condition had also accessed other treatment, this would still be a systematic, post-randomization removal of participants. However, it is easier to develop alternate explanations of the findings of this trial because all participants using other treatment were from the control condition.

From one perspective, it is possible that the excluded participants were those who were experiencing the most serious problems with their Cannabis use. If this is the case, it could make it less likely that significant differences would be observed between experimental condition by reducing the variance between participants. Alternatively, perhaps these were the five participants in the control condition who were the most motivated to reduce their Cannabis use. This could mean that participants in the control condition were less motivated, on average, than those in the intervention condition, to do something about their Cannabis use. This could serve as an alternate explanation of the findings in this study.

Given that only five participants were removed out of 225 (or 230 if control and intervention group participant totals are added?) randomized at baseline, it is quite possible that this alternate explanation is untrue. However, the authors do expose themselves to alternate explanations of the findings by the removal of these participants. If the systematic removal of participants post-randomization is deemed necessary, one possible solution would be to run (and report on) the analysis with and without these participants included. Such sensitivity analyses would go a long way towards addressing any possible confounds that may have been introduced. 
